# Body size and vocalization in primates and carnivores

**DOI:** 10.1038/srep41070

**Published:** 2017-01-24

**Authors:** D. L. Bowling, M. Garcia, J. C. Dunn, R. Ruprecht, A. Stewart, K.-H. Frommolt, W. T. Fitch

**Affiliations:** 1 Department of Cognitive Biology, University of Vienna, Vienna, Austria; 2 L’Equipe de Neuro-Ethologie Sensorielle, Université de Lyon/Saint Etienne, Saint Étienne, France; 3 Department of Archaeology & Anthropology, University of Cambridge, Cambridge, UK; 4 Animal and Environment Research Group, Anglia Ruskin University, Cambridge, UK; 5 Center for Language Evolution, University of Edinburgh, Edinburgh, UK; 6 Museum für Naturkunde Berlin, Leibniz Institute for Evolution and Biodiversity, Berlin, Germany.

## Abstract

A fundamental assumption in bioacoustics is that large animals tend to produce vocalizations with lower frequencies than small animals. This inverse relationship between body size and vocalization frequencies is widely considered to be foundational in animal communication, with prominent theories arguing that it played a critical role in the evolution of vocal communication, in both production and perception. A major shortcoming of these theories is that they lack a solid empirical foundation: rigorous comparisons between body size and vocalization frequencies remain scarce, particularly among mammals. We address this issue here in a study of body size and vocalization frequencies conducted across 91 mammalian species, covering most of the size range in the orders Primates (n = 50; ~0.11–120 Kg) and Carnivora (n = 41; ~0.14–250 Kg). We employed a novel procedure designed to capture spectral variability and standardize frequency measurement of vocalization data across species. The results unequivocally demonstrate strong inverse relationships between body size and vocalization frequencies in primates and carnivores, filling a long-standing gap in mammalian bioacoustics and providing an empirical foundation for theories on the adaptive function of call frequency in animal communication.

Pioneering work on the biology of vocal communication focused on comparative anatomy and physiology, proposing a primary role for the larynx in protecting the respiratory system and suggesting how this arrangement inevitably led to the production of sounds imbued with valuable information about the biological status of the vocalizer^1^,^2^,^3^. More recent work has added a focus on the acoustics of vocalizations themselves, leading to further hypotheses about the communicative potential of specific acoustic properties^4^,^5^. One basic aspect of the modern approach is *size-frequency allometry*: the study of the relationship between the size of an animal’s body and the frequency content of its vocalizations. Morton (1977) was among the first to formally propose a link between these domains^6^. In his widely cited motivation-structural rules hypothesis, he proposed that vocalization frequency was shaped by its capacity to communicate biologically critical information about body size^6^,^7^. The key assumption underlying this idea is that larger animals inevitably produce vocalizations with lower frequency content than smaller animals. This *negative* size-frequency allometry provided the physical foundation upon which early biological associations were built, ultimately channeling the evolution of vocalization under pressure from natural selection and resulting in a widely conserved size-frequency code in animal communication.

General support for Morton’s assumption comes from our modern understanding of the physics of vocal production (Fig. 1). At the broadest level, vocalizations can be described as the result of tissue vibrations generated by the passage of air through a constriction in an animal’s vocal tract. In most tetrapods (excluding birds) the principal oscillator is the vocal folds within the larynx. During phonation, air from the lungs passes between the vocal folds, setting them into oscillation and producing a rich spectrum of vibrations that provide the acoustic power for vocalization. When the pattern of vocal fold vibration is approximately periodic, the resulting spectrum is harmonic and we perceive a voice pitch related to its fundamental frequency (‘F0’), which corresponds to the rate of one open/close cycle of vocal fold motion. These laryngeal “source” vibrations are propagated into the air in the supralaryngeal vocal tract, whose resonances act as acoustic “filters”, imposing a second distinct set of resonant frequencies called formants on the laryngeal spectrum^8^,^9^.

Two facts about this combination of source and filter constrain the frequency content of vocalization. First, the lowest F0 at which the vocal folds can vibrate is fundamentally limited by their length: longer folds vibrate at lower F0s^9^. Second, the lowest frequency at which the air within the vocal tract can resonate is also fundamentally limited by its length: longer tracts generate lower formants^8^,^9^. Thus, to the extent that vocal fold and vocal tract length scale in proportion to body size, the vocalizations of larger animals can, based on physical principles, be expected to comprise lower frequencies than the vocalizations of smaller animals, as Morton assumed.

Given this physical backdrop, it is somewhat surprising that empirical evidence for negative size frequency allometry is rather sparse, particularly among mammals. Traditionally, the best evidence has come instead from comparative studies of birds and frogs. For example, Wallschläger (1980) found evidence for negative allometry between body mass and center frequency (the geometric mean of a spectrum) across a sample of 90 European passerine bird species (*R*^*2*^ = 0.59)^10^. Likewise, Gingras *et al*.^11^ found evidence for negative allometry between body length and F0 across a sample of 136 frog species (*R*^*2*^ = 0.57)^11^. In both of these studies, the inclusion of species exhibiting a wide range of body sizes (~5–1400 g in^10^ and 14–155 mm in^11^) appears to have been critical. Studies examining narrower ranges of body sizes, e.g., within a single species, have produced mixed results, finding evidence for negative allometry in some cases^12^,^13^,^14^ but not others^15^,^16^,^17^,^18^.

The principal challenge in conducting large-scale interspecific studies of size-frequency allometry is characterizing the vocal behavior of each target species in a clear and consistent way^19^. The approach taken by most anuran and avian studies has been to limit the analysis to a single *call type* that can be reliably identified across species, sacrificing representativeness in favor of comparability. Wallschäger (1980), for example, focused on male “song” in birds, whereas Gingras *et al*.^11^ focused on male frog “advertisement calls”^10^,^11^. Unfortunately, this approach is not easily adapted to comparably diverse samples of mammals, where interspecific variability in vocal behavior is arguably greater and vocal repertoires often exhibit acoustically graded rather than discrete structures^19^,^20^,^21^. Both of these features complicate the definition and identification of clear call types as a basis for comparison, preventing straightforward extension of the avian and anuran approach to mammals.

To date, we are aware of only three studies that have attempted to derive interspecific size-frequency allometry in mammals. Jones (1996) found evidence for negative allometry between body mass and the frequency of maximal amplitude in bat echolocation calls (no *R*^*2*^ reported)^22^. More relevant here, Hauser (1993) found evidence for negative allometry between body mass and a parameter he referred to as “mean repertoire frequency” in a sample of 36 primate species (*R*^*2*^ = 0.54)^23^. Finally, in the most comprehensive study to date Charlton & Reby (2016) found evidence for negative allometry between body mass and F0 in a sample of 67 terrestrial mammal species (*R*^*2*^ = 0.59), as well as between body mass and formant frequency spacing in a smaller sample of 35 terrestrial mammal species (*R*^*2*^ = 0.58)^5^. Although these studies provide an important foundation for the study of mammalian size-frequency allometry, the accuracy of the relationships derived is limited by the way in which vocal behavior has been characterized across species. Hauser (1993)’s approach, for example, was to include everything he could obtain from published literature, provided that each included species had at least 5 described call types. Although reasonable at the time, this approach is problematic in that the number of vocalizations included for each species was highly variable (between 5–40), calling into question the validity of interspecific comparisons between mean values. A related point is that because original audio recordings were not typically obtained, the capacity for clear and consistent frequency measurements was inherently limited (mean repertoire frequency thus represented a poorly defined mixture of F0 and various frequencies of maximal amplitude, often estimated by visual inspection of published spectrograms). Finally, Hauser’s regression analyses were not phylogenetically controlled, making it likely that the reported relationships are distorted by statistical codependence between closely related species^24^. Charlton & Reby (2016) were considerably more careful, focusing on well-defined acoustic properties, conducting thorough phylogenetic analyses, and going further to incorporate data on habitat and sexual dimorphism. Even in this impressive study, however, the fact that the majority of the acoustic data was compiled from third-party sources may have introduced inconsistencies in measurement across species as well as variation in the representativeness of mean values.

The present study was designed to address these limitations as well as the general paucity of size-frequency data in mammals. We compiled data representing 91 mammalian species, including 50 primates and 41 carnivorans (hereafter ‘carnivores’) (Table 1). The selected species cover a wide range of body sizes – from the pygmy marmoset (*Cebuella pygmaea*; 110 g) to the Western gorilla (*Gorilla gorilla*; 120 Kg), and from the least weasel (*Mustela erminea*; 140 g) to the polar bear (*Ursus maritimus*; 250 Kg) – maximizing the likelihood of observing the effects of anatomical constraints on vocal production. In contrast with previous work, our method of obtaining vocalization data is focused on using original recordings for all species, taking full advantage of modern improvements in high-throughput computerized analysis, digital signal processing and the availability of digital recordings through online databases. We use a novel algorithm to capture spectral variability and standardize vocalization selection, giving rise to data specifically prepared for interspecific comparison. These data are compared to body size data using both traditional and phylogenetically controlled regression techniques, resulting in the derivation of empirical size-frequency allometry in primates and carnivores.

## Materials and Methods

### Body size data

The measure of body size used in the comparisons presented here is body length, defined as “head + body” length, which excludes tail length and refers to the distance between the ischium of the pelvis and the tip of the snout in carnivores, or the top of the skull in primates (also known as “crown-rump” length). With the exception of California sea lions (*Zalophus californianus*) and humans, all body size data were obtained from the “Handbook of the Mammals of the World”^25^,^26^. This source typically reports length ranges (separated by sex for about 1/3 of the species in our sample). Ranges were converted to means, by species or by sex then species as required. For sea lions, mean body length was calculated from sex-specific range data in^27^; for humans, mean body length was calculated by multiplying sex-specific standing height values by average “sitting-ratios” as provided in^28^.

### Vocalization database

With the exception of humans and Bolivian red howler monkeys, all vocalization recordings were compiled either from the Animal Sound Archive at the Museum für Naturkunde Berlin, or commercially available CDs^29^,^30^,^31^,^32^,^33^,^34^,^35^,^36^,^37^,^38^. Recordings for which there was information indicating that the vocalizing animal was not an adult were excluded from the database. The human vocalizations consisted of emotionally expressive speech, uttered by male and female speakers of multiple languages^39^. The Bolivian red howler vocalizations were obtained by JCD in collaboration with the Senda Verde Animal Refuge in Bolivia. The resulting database comprised ~13.5 hours of digital audio stored in 816 audio files (.WAV or.AIFF format). Depending on the quality of the original recordings, sampling rates were either 44.1, 48 or 96 kHz, and bit depths were 16-, 24- or 32-bits.

### Preprocessing and vocalization selection

Prior to vocalization selection, each of the 816 audio files was automatically segmented using a custom Matlab (version R2015a; Nantick, MA) script that identified temporally contiguous segments of supra-threshold intensity (intensity was defined by low pass filtering rectified audio waveforms at 5 Hz using a 3^rd^ order Butterworth filter; threshold was defined as >5% of the maximum intensity in a given file). A margin of 100 ms was used, such that segment start times were placed 100 ms prior to when intensity rose above threshold, and end times were placed 100 ms after when intensity fell back below threshold. If either of these 100 ms margins entered into another suprathreshold region, the regions were treated as contiguous and extracted as a single segment. This procedure resulted in 6527 audio segments, each of which was subsequently sorted into a “low” or “high” signal-to-noise ratio category on the basis of manual aural and visual inspection in Praat^40^. Low signal-to-noise segments – characterized by high-energy environmental noise, the presence of multiple individual vocalizers with overlapping spectra, and/or sounds other than the targeted vocalizations (e.g., cage-rattling) – accounted for 52% of segments overall and were excluded from further analysis. These preprocessing steps resulted in a total of 3151 segments with high signal-to-noise ratios, each representing a single temporally discrete vocalization. The determination of which vocalizations to include in further analyses was performed by a novel three-step selection algorithm also implemented in Matlab. First, the spectrum of each vocalization was determined by multiplying the entire segment by a Hamming window and computing a single discrete Fourier transform (frequency resolution was determined by the number of samples in a given segment, Mean = 3.18 Hz, SD = 4.77 Hz). Second, the frequency of maximum amplitude in each spectrum was identified and used as the basis for sorting the vocalizations of each species into an ordered list. Third, the sorted vocalization list was used to define a maximally-spaced selection of 10 vocalizations. For example, in a list of 100 sorted vocalizations, those selected would be in list positions 1, 12, 23, 34, 45, 56, 67, 78, 89 and 100. This algorithm provides a standardized approach to capturing the entire spectral variability range present in a given species’ vocalizations. Systematically applying it here ensured that the vocalization data selected to represent each species was derived in precisely the same manner, maximizing the validity of subsequent interspecific comparisons. All in all, following this procedure resulted in a subset of 910 vocalizations (10 per species) selected for more detailed frequency analysis.

### Frequency analysis

Our analysis of vocalization frequencies focused on dominant frequency (or “DF”) and F0. DF is simply defined as the frequency of maximum amplitude in the spectrum of a vocalization, and was thus already determined during the selection process described above. Nevertheless, for each of the 910 vocalizations in our subset, the DF value ca.lculated by Matlab was manually verified in a second (more detailed) aural and visual inspection in Praat. If the DF value assigned by Matlab did not match acoustic energy present in the vocalization (e.g., because it corresponded to background noise instead), that vocalization was replaced with the neighboring vocalization from the species’ sorted list (this was necessary for 53 of the 910 selected vocalizations; ~6%). Once the accuracy of all DF values was confirmed, a ‘DF10’ value was calculated for each species, corresponding to the mean DF calculated across all 10 of their selected vocalizations.

**Figure 1:**
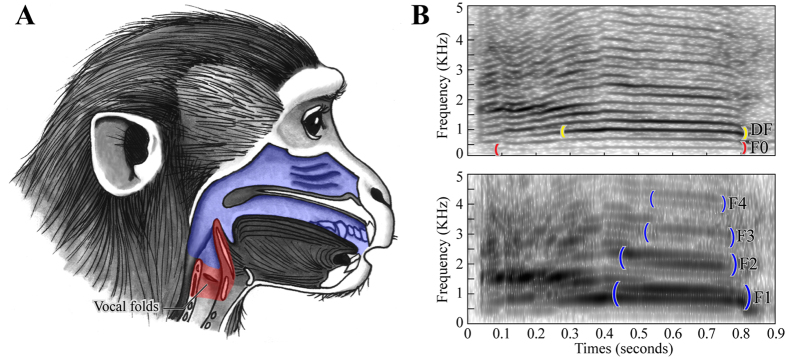
Vocal production in mammals. (**A**) Cut-away diagram of a macaque monkey showing the vocal production apparatus. The larynx is highlighted in red, the supralaryngeal vocal tract is highlighted in blue. (**B**) Spectrograms depicting key frequency features of animal vocalization. Both spectrograms show the same vocalization (a snow leopard contact call). In the top panel, longer temporal analysis windows (0.25 seconds) emphasize the fundamental frequency (‘F0’; bracketed in red) and its harmonics (horizontal black lines). The dominant frequency (‘DF’; bracketed in yellow) is also labeled. In the bottom panel, shorter temporal analysis windows (0.006 seconds) emphasize the first four formants (‘F1-4’; bracketed in blue).

In contrast to DF, which can be measured for any vocalization, F0 can only be measured for vocalizations that are produced by regular vocal fold vibration. Spectrally, the defining characteristic of these “tonal” vocalizations is the presence of clear harmonics (see Fig. 1B). The only exceptions are “pure tone” vocalizations, which exhibit focused energy at a single specific frequency and do not possess harmonics. For each of the 910 selected vocalizations, those that were harmonic or pure tone were identified for F0 measurement during the second aural and visual inspection in Praat. Following identification, F0 measurements were made using a manually supported algorithmic approach. First, a visual estimate of the frequency distance between harmonics was made from the spectrogram and used to initialize the parameters of Praat’s “To Pitch” algorithm (default values are defined for human speech and thus often needed to be adjusted; this was most often the case for “Pitch range”, followed by “Voicing threshold”, “Silence threshold”, “Octave cost”, “Voiced/unvoiced cost” and “Octave jump cost”). Second, these parameters were adjusted until the F0 values identified by the algorithm matched the estimate and corresponded to the first harmonic observed in the spectrogram. When visual and algorithmic methods could not be made to agree, or when harmonics were too vague or complex to estimate their spacing (e.g., in cases of bifurcation or subharmonics^41^), F0 was not measured. Following this procedure, F0 could be measured in 664 of the 910 selected vocalizations (~73%). In order to avoid differences in the number of vocalizations used to calculate means across species, comparisons involving F0 were limited to those species that had at least six vocalizations with measured F0 values. Using 6 vocalizations as our criterion allowed us to include 74 species in these analyses (41 primates and 33 carnivores). Each of these species was represented by an ‘F06’ value, corresponding to the mean F0 calculated across six vocalizations. To allow fair comparisons of size-frequency relationships based on DF and F0, ‘DF6’ values (also calculated on the basis of 6 vocalizations) were also determined for these 74 species. Which vocalizations to include in the DF6 and F06 calculations was determined using the same maximally-spaced selection technique described above for vocalization selection. For DF6, the ordered list always included all 10 vocalizations per species and was sorted from lowest to highest by DF; for F06, the starting list included however many of a species’ vocalizations that had F0 values and was sorted from lowest to highest F0.

### Statistical analyses

Two types of linear regression analysis were used to model relationships between body length and vocalization frequencies: (1) an ordinary least squares (OLS) regression; and (2) a bisector regression based on the best supported of 5 different generalized least squares (GLS) models. Both approaches have distinct advantages and disadvantages here. The advantages of OLS are that it allows for the use of standard statistical tests (e.g., *R*^*2*^, *F*-test, *ANCOVA*) as well as direct comparison with other/older studies. The disadvantages are that it fails to control for the potential statistical codependence of data points representing closely related species^24^,^42^, and that it only minimizes error with respect to one of the two variables in a given comparison. This situation is reversed for the bisector regressions, which combine phylogenetic techniques to evaluate and control for statistical codependence between closely related species^43^, and bisector techniques that account for error in both variables (appropriate here because both body length and vocalizations frequencies are measured and thus subject to error^44^), but for which standard statistical tests like *R*^*2*^ and *ANCOVA* do not apply^43^. Following Charlton & Reby (2016), the GLS models examined here include one non-phylogenetic model (NP), and four phylogenetic models, each of which tests a different assumption about the evolutionary process based on phylogenetic tree data^45^ (shown in Supplementary Figs 1 and 2). The phylogenetic models were a pure Brownian motion model (BM), a Brownian motion + Pagel’s Lambda model (BM + λ), a Brownian motion + Grafen’s Rho model (BM + ρ), and an Ornstein-Uhlenbeck model (OU)^5^ (see Supplementary Text S1 for further details).

OLS regressions were calculated in R using the ‘stats’ package function ‘lm.m’^46^. GLS regressions were calculated in R using the ‘nlme’ package function ‘gls’^47^ in connection with ‘APE’ package correlation structures^48^. Correlation structure was set to ‘NULL’ for NP models, ‘corBrownian’ for BM models, ‘corPagel’ for BM + λ models, ‘corGrafen’ for BM + ρ models, and ‘corMartins’ for OU models. As a heuristic indicator of model support, we calculated Akaike Information Criteria corrected for sample size (AICc; R package ‘AICmodavg’ function ‘AICc’)^49^. The model with the lowest AICc was selected for bisector calculation, which was carried out in Matlab according to the procedure described in Supplementary Text S1. Differences between OLS regressions for primates and carnivores were evaluated for statistical significance using ANCOVA performed in Matlab (function ‘aoctool.m’). Finally, all variables were checked for normality prior to comparisons using Shapiro-Wilk tests performed in Matlab (function ‘swtest.m’). Log-transforms (base-10) were necessarily to achieve normality for body length, DF10, F06, and DF6. These transformations also improved the fits of linear regression.

## Results

**Figure 2:**
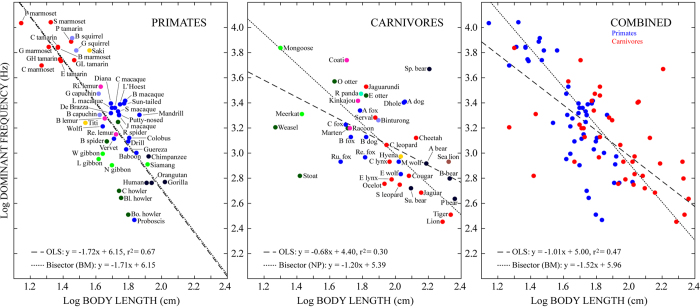
Body length and dominant frequency. The base-10 logarithm of mean body length plotted against the base-10 logarithm of mean dominant frequency (‘DF10’) for 50 primate species (left), 41 carnivore species (middle), and all 91 species combined (right). In the left and middle panels, color represents biological family (legend in Fig. 4) and each species is labeled with an abbreviated form of its common name (full names in Table 1); in the right panel primates are shown in blue and carnivores in red. Dashed lines depict ordinary least squares (OLS) regressions; dotted lines depict bisector regressions (equations at lower left). Primate and combined bisector regressions were based on phylogenetic Brownian motion (BM) models; carnivore bisector regressions were based on non-phylogenetic (NP) models. Statistics for regression analyses are given in Table 2A (see Supplementary Table S1a for AICc values of GLS models).

The results of comparing the logarithm of mean body length (‘logBL’) with the logarithm of mean dominant frequency (‘logDF10’) for all 91 species are shown in Fig. 2 and Table 2A. All regressions showed significant negative relationships for primates, carnivores, and both orders combined, providing empirical verification of negative size-frequency allometry within and across these mammalian orders. Similar results were obtained when the sample size was reduced to 74 species and DF10 was replaced with DF6 (Table 2B; Supplementary Fig. S3). In addition to these principle findings regarding negative size-frequency allometry, several interesting differences were apparent between primates and carnivores. First, the *R*^*2*^ values of the OLS regressions show that LogBL explained over twice as much of the variance in logDF10 for primates than for carnivores (67% and 30% respectively). The proportion of variance explained by the carnivore OLS regression could be improved considerably by excluding the two greatest outliers (spectacled bear and stoat; raising *R*^*2*^ to 0.50) but we know of no *a priori* reason to exclude these species and reexamination of their data confirmed the accuracy of their exceptional status. Together with the overall greater dispersion of the carnivore data, these results show that although the DF of a vocalization is generally a good predictor of body length, the relationship is much stronger for primates than it is for carnivores. Second, a significant difference between primates and carnivores was also found in OLS regression slopes (*β*_*prim*_ = −1.72 vs. *β*_*carn*_ = −0.68; ANCOVA LogBL x order *F*_1,87_ = 18.37, *P* < 0.0001), such that DF drops much more rapidly with increasing body length for primates than for carnivores. Examination of the bisector regressions, however, indicated that much of this second difference (~50%) is attributable to limitations of OLS regression. While the primate bisector regression (based on BM models) was nearly identical to its OLS counterpart, the carnivore bisector regression (based on NP models) was considerably steeper, resulting in a much smaller difference in slope between orders (*β*_*prim*_ = −1.71 vs. *β*_*carn*_ = −1.20).

**Figure 3:**
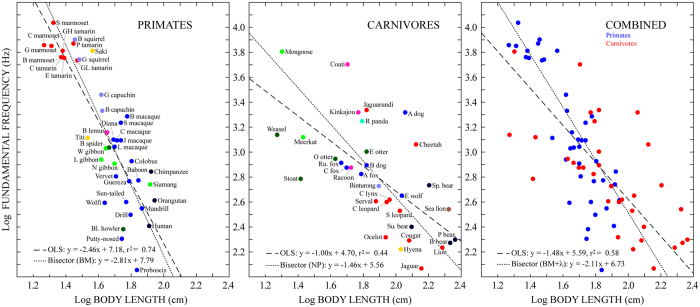
Body length and fundamental frequency. The base-10 logarithm of mean body length plotted against the base-10 logarithm of mean fundamental frequency (‘F06’) for 41 primate species (left), 33 carnivore species (middle), and all 74 species combined (right). In the left and middle panels, color represents biological family (legend in Fig. 4), and each species is labeled with an abbreviated form of its common name (full common names and Latin names in Table 1); in the right panel all primates are shown in blue and all carnivores in red. Dashed lines depict ordinary least squares (OLS) regressions; dotted lines depict bisector regressions (equations at lower left). Primate bisector regressions were based on phylogenetic Brownian motion models; carnivore bisector regressions were based on non-phylogenetic (NP) models; combined bisector regressions were based on phylogenetic Brownian motion + Pagel’s Lambda (BM + λ) models. Statistics for regression analyses are given in Table 3 (see Supplementary Table S2 for AICc values of GLS models).

The results of comparing logBL with the logarithm of mean fundamental frequency (‘logF06’) for the subset of 74 species are shown in Fig. 3 and Table 3. As in the previous analysis, all regression analyses between logBL and logF06 showed significant negative relationships for primates, carnivores, and both orders combined, providing further empirical evidence for negative size-frequency allometry within and across these mammalian orders. Also in parallel with the previous results, a difference between primates and carnivores was observed in the *R*^*2*^ values of the OLS regressions, such that the proportion of variance in logF06 explained by logBL was higher for primates than for carnivores (74% and 44% respectively). Finally, the primate OLS regression was determined to be significantly steeper than the carnivore OLS regression (*β*_*prim*_ = −2.46 vs. *β*_*carn*_ = −1.00; ANCOVA: LogBL x order *F*_1,70_ = 21.48, *P* < 0.0001). Unlike the DF analyses, however, the magnitude of this slope difference was not much decreased between the bisector regressions (again based on BM models for primates and NP models for carnivores), which exhibited a comparable slope difference (*β*_*prim*_ = −2.81 vs. *β*_*carn*_ = −1.46). These results thus provide strong evidence that F0 drops more rapidly with increasing body length for primates than for carnivores.

**Figure 4:**
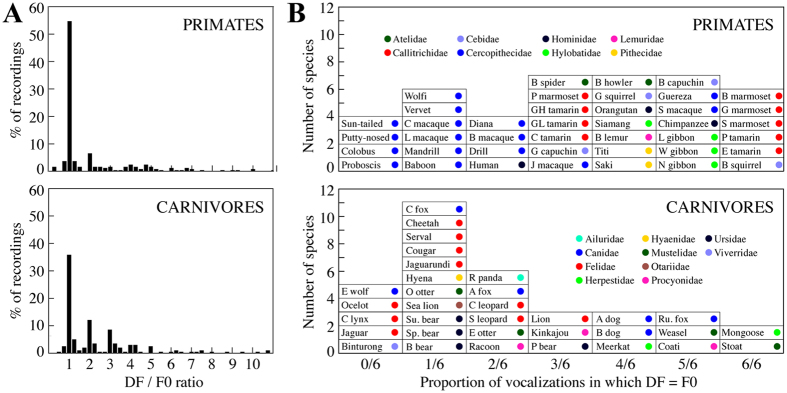
DF/F0 ratios. (**A**) Histograms of the DF/F0 ratios for each of the vocalizations used to calculate the F06 variable (n = 444) for 41 primate species (top) and 33 carnivore species (bottom). Histogram bins run from −0.125 to 11.125 in steps of 0.25. (**B**) The proportion of vocalizations (out of 6) in which DF/F0 ≈ 1 broken down by species.

Aside from the differences between primates and carnivores, there were also clear differences between allometric relationships derived using DF and F0. Using F0 resulted in better regression fits and steeper slopes across all comparisons (cf. Figs 2, 3; see also Supplementary Fig. S3). Overall, F0 values tended to be lower than DF values, resulting in a downward shift of many of the data points in comparisons involving F0 relative to DF. This was particularly true for larger species, giving rise to steeper slopes for the F0 regressions. To examine the relationship between DF and F0 more closely, we calculated the ratio between DF and F0 for the subset of vocalizations used to calculate the F06 values (because these vocalizations had both F0 and DF values). The results of this analysis are shown in Fig. 4A. In accordance with the downward shift of data points observed in the F0 analyses, the DF/F0 ratio was found to be ≥1 for most vocalizations (95% for primates and 97% for carnivores). DF and F0 were approximately the same (i.e., DF/F0 ≈ 1) in 55% of the primate vocalizations and 36% of the carnivore vocalizations. When DF and F0 were not the same, DF corresponded to a harmonic multiple of F0 in a further 16% of the primate vocalizations and 28% of the carnivore vocalizations. These results show that F0 is frequently the most powerful component of mammalian vocalization (particularly for primates), and suggest that when it is not, vocal tract resonances may play an important role in determining the most powerful frequency content (see Fig. 1B).

The proportion of calls in which DF and F0 were the same is broken down by species in Fig. 4B. The prevalence of relatively large species towards the left-hand side and small species towards the right-hand side of these graphs suggests that the proportion of vocalizations in which DF/F0 = 1 may be related to body size. This possibility was tested by examining the correlation between body length and proportion of vocalizations in which DF/F0 = 1. The results indicated highly significant relationships for both primates and carnivores *(Spearman’s r* = −0.48, *P* = 0.0014, and *r = *−0.50, *P* = 0.0031 respectively). These results show that for the species considered here, the smaller an animal is the more likely it is that F0 will be the most powerful frequency component of their vocalizations.

## Discussion

The results of the analyses presented here provide clear empirical verification of negative size-frequency allometry in primates, and the first empirical evidence for negative size-frequency allometry specifically in carnivores. Whether based on DF or F0, the relationships between primate body size and vocalization frequencies were considerably stronger than those previously reported (*mean β = *−2.18, *mean R*^*2*^ = 0.71 vs. for example *β = *−0.39, *R*^*2*^ = 0.54 in ref. 23), demonstrating advantages of our more rigorous methodology and showing that body size is even more closely related to vocalization frequencies in this order than previously shown. Intriguingly, these same relationships were much weaker for carnivores (*mean β = *−1.09, *mean R*^*2*^ = 0.37), suggesting that the capacity of fundamental and/or dominant frequency to signal body size in vocal communication should not be assumed to be equal across mammalian orders and should be examined independently in different clades whenever possible.

Focusing on the combined analyses of primates and carnivores, the relationship between body length and F0 derived here (see Fig. 3) can be compared with that between body mass and F0 derived in Charlton & Reby (2016), albeit with a different set of mammalian species (overlap = 12 primates and 4 carnivores)^5^. These relationships had very similar *R*^*2*^values (0.58 here vs. 0.59 in ref. 5), but the slopes of our regressions were considerably steeper (*mean βs* = −1.8 here vs. −0.5 in ref. 5). This remained true even when comparing the exact same regression model: the BM + λ regression was the best-supported model for F0 comparisons in both studies; prior to bisector calculation the *β* value for our model was −1.18 ( ± 0.23 SE; see Table 3) whereas the comparable *β* value in ref. 5 was −0.5 (±0.09 SE). While it is plausible that this difference was driven, at least in part, by our novel approach to collecting vocalization data, additional differences (e.g., in the index of body size and/or the species sampled) are also likely to have contributed.

Our results also show that, despite a strong tradition of using DF as the acoustic parameter in studies of size-frequency allometry (e.g., refs 15, 22, 23), F0 performs much better than DF at predicting body size among mammals. Further examination of the relationship between DF and F0 showed that these parameters are more likely to be the same in primate than carnivore vocalizations, and more likely to be the same in the vocalizations of smaller than larger species in both clades. The latter point is consistent with the fact that smaller species produce higher F0s. Higher F0s result in more widely spaced harmonics that are less likely to be emphasized by a vocal tract resonance.

Although it is presently unclear why the size-frequency relationships determined here are stronger for primates than carnivores, at least three possibilities come to mind. The first is that this finding may be partially artifactual. Carnivores have typically received less research attention than primates, and it is possible that the vocalization data compiled to represent them here somehow reflects this neglect. While our approach ensured equal representation for each species in the subset of selected vocalizations, it is possible that the vocal behavior of carnivores was less adequately represented in the original database from which the subset was selected. We did not, however, find any evidence in support of this possibility. Examination of the average number of high SNR vocalizations available for each species after preprocessing suggested better representation for primates (*mean* = 126, *SD* = 134) than carnivores (*mean* = 85, *SD* = 58), but this difference was not significant (*Mann-Whitney U* = 2494, *n*_1_ = 41, *n*_2_ = 50*, P* = 0.12). Nevertheless, it remains possible that there were differences between orders in how well the selected vocalizations represented the vocal ranges of the species in question.

A second possibility is that the differences observed here between primates and carnivores reflect real ecological differences between these clades, for example in habitat, social structure, diet and/or lifestyle^5^. Although within-clade variation for any one of these dimensions is quite large, variation between clades is likely to be larger, and thus could plausibly support important differences in the selective pressures acting on vocal production and behavior in these groups.

A final possibility, not mutually exclusive with the second, is that weaker size-frequency allometry in carnivores reflects greater variability in vocal behavior. Some evidence in support of this possibility is that the average number of non-tonal vocalizations in our selected subset was significantly greater for carnivores (*mean* = 3.2/10, *SD* = 2.1) than for primates (2.3/10; *SD* = 2.6; *Mann-Whitney U* = 2021.5, *n*_1_ = 41, *n*_2_ = 50*, P* = 0.02). The more frequent occurrence of non-tonal vocalizations in carnivores indicates less uniformity in acoustic structure and, potentially, a greater reliance on nonlinear modes of vocal production. While either of these factors would contribute noise to comparisons between DF and body length, they would presumably not affect comparisons between F0 and body length. The relative weakness of this later relationship in carnivores therefore remains a puzzle.

The influence of different modes of vocal production also provides a plausible answer to the question of why the size-frequency relationships established here were stronger for F0 than DF. The DF of a vocalization is ambiguously determined through the interaction of multiple vocal production mechanisms. For example, if F0 is the frequency of maximal amplitude, DF will correspond the lowest rate of vocal fold vibration; but if the frequency of maximal amplitude corresponds to the peak of a formant resonance, or a particular harmonic near that peak, DF will correspond instead to a particular vocal tract configuration, or an interaction between both vocal fold vibration and vocal tract configuration. The fact that DF is not directly related to any single aspect of vocal anatomy limits its usefulness in allometric comparisons (but not necessarily acoustic communication) because it cannot be expected to vary systematically with any single measurement. Using DF is thus likely to introduce noise into allometric comparisons. Using F0 instead addresses this problem because the F0 of a vocalization is determined by the rate of vocal fold vibration and can thus be expected to vary systematically with vocal fold length^9^. In addition to DF and F0, it should be noted that the spacing of formant frequencies is an important acoustic indicator of body size that was not examined here^5^,^50^. An assessment of formant frequencies across the variety of species and vocalizations included here was complicated by the fact that accurate measurement of formants typically requires calls with broadband source vibration^51^, which was only the case for a small subset of our vocalizations (and disproportionately from larger animals).

A final point concerns species that appeared as consistent outliers in both DF and F0 regressions. Among primates, these species included the howler monkeys (*Alouatta caraya, A. seniculus* and *A. sara*), the proboscis monkey (*Nasalis larvatus*) and the putty-nosed monkey (*Cercopithecus nictitans*), all of which exhibited lower DF and/or F0 values than expected on the basis of their body lengths (see Figs 2 and 3). In the case of the howler monkeys, these differences can be understood in terms of well-documented laryngeal hypertrophy^52^,^53^. In the case of the proboscis monkey, an elongated nasal appendage may provide an explanation for exceptionally low DF values, but the causal basis for their exceptionally low F0 values as well as those of the putty-nosed monkey remains unknown. Among carnivores, consistently outlying species included the spectacled bear (*Tremarctos ornatus*) and the African hunting dog (*Lycaon pictus*), which exhibited higher vocalization frequencies than predicted on the basis of body length, as well as the least weasel (*Mustela nivalis*) and the stoat (*Mustela erminea*), which exhibited lower vocalization frequencies than predicted. We are unaware of any documented specializations in the vocal anatomy of these species. The anatomical factors underlying their exceptional DF and F0 values may thus represent opportunities to further our understanding of biological adaptation in vocal communication.

In conclusion, the results presented here provide unequivocal empirical evidence of negative size-frequency allometry in mammals using novel methods to derive clear and consistent vocalization data across primates and carnivores. In accordance with the founding assumption of Morton’s motivation-structural rules hypothesis^6^, these results suggest that across a wide range of body sizes both the dominant and fundamental frequency of vocalizations are inherently related to the size of vocalizers, implying that biological associations based on these relationships are indeed likely to be adaptive. Thus, as for the other clades examined so far, for primates and carnivores negative size-frequency allometry appears to be a fundamental aspect of the bio-acoustic landscape. However, our results also suggest caution in applying the details of any specific relationship too broadly: even in the phylogenetically limited set of mammalian species considered here, large differences were apparent between biological orders.

## Additional Information

**How to cite this article**: Bowling, D. L. *et al*. Body size and vocalization in primates and carnivores. *Sci. Rep.* **7**, 41070; doi: 10.1038/srep41070 (2017).

**Publisher's note:** Springer Nature remains neutral with regard to jurisdictional claims in published maps and institutional affiliations.

## Supplementary Material

Supplementary Information

## Figures and Tables

**Table 1:** Abbreviations, common names and Latin names for all 50 primates species and all 41 carnivore species.

#	Abbreviation	Common Name	Latin Name	#	Abbreviation	Common Name	Latin Name
**A. Primate Species**							
1	B capuchin	Brown capuchin	*Sapajus apella*	26	Guereza	Mantled guereza	*Colobus guereza*
2	B lemur	Black-and-white ruffed lemur	*Varecia variegata*	27	Human	Human	*Homo sapiens*
3	B macaque	Barbary macaque	*Macaca sylvanus*	28	J macaque	Japanese macaque	*Macaca fuscata*
4	B marmoset	Buffy-headed marmoset	*Callithrix flaviceps*	29	L gibbon	Lar gibbon	*Hylobates lar*
5	B spider	Black-headed spider monkey	*Ateles fusciceps*	30	L macaque	Lion-tailed macaque	*Macaca silenus*
6	B squirrel	Black-capped squirrel monkey	*Saimiri boliviensis*	31	L’Hoest	L’Hoest’s monkey	*Allochrocebus lhoesti*
7	Baboon	Hamadryas baboon	*Papio hamadryas*	32	Mandrill	Mandrill	*Mandrillus sphinx*
8	Bl. howler	Black howler	*Alouatta caraya*	33	N gibbon	Northern white-cheecked gibbon	*Nomascus leucogenys*
9	Bo. howler	Bolivian red howler	*Alouatta sara*	34	Orangutan	Orangutan	*Pongo pygmaeus*
10	C howler	Colombian red howler	*Alouatta seniculus*	35	P marmoset	Pygmy marmoset	*Cebuella pygmaea*
11	C macaque	Crested macaque	*Macaca nigra*	36	P tamarin	Pied tamarin	*Saguinus bicolor*
12	C marmoset	Common Marmoset	*Callithrix jacchus*	37	Proboscis	Proboscis monkey	*Nasalis larvatus*
13	C tamarin	Cotton-top tamarin	*Saguinus oedipus*	38	Putty-nosed	Putty-nosed monkey	*Cercopithecus nictitans*
14	Chimpanzee	Chimpanzee	*Pan troglodytes*	39	R spider	Red-faced spider monkey	*Ateles paniscus*
15	Colobus	Black colobus	*Colobus satanas*	40	Re. lemur	Red-ruffed lemur	*Varecia rubra*
16	De Brazza	De Brazza’s monkey	*Cercopithecus neglectus*	41	Ri. Lemur	Ring-tailed lemur	*Lemur catta*
17	Diana	Diana monkey	*Cercopithecus diana*	42	S macaque	Stump-tailed macaque	*Macaca arctoides*
18	Drill	Drill	*Mandrillus leucophaeus*	43	S marmoset	Silvery marmoset	*Mico argentatus*
19	E tamarin	Emperor tamarin	*Saguinus imperator*	44	Saki	White-faced saki	*Pithecia pithecia*
20	G capuchin	Guianan weeper capuchin	*Cebus olivaceus*	45	Siamang	Siamang	*Symphalangus syndactylus*
21	G marmoset	Geoffroy’s tufted-ear marmoset	*Callithrix geoffroyi*	46	Sun-tailed	Sun-tailed monkey	*Allochrocebus solatus*
22	G squirrel	Guianan squirrel monkey	*Saimiri sciureus*	47	Titi	Red-bellied titi	*Callicebus moloch*
23	GH tamarin	Golden-headed lion tamarin	*Leontopithecus chrysomelas*	48	Vervet	Vervet monkey	*Chlorocebus pygerythrus*
24	GL tamarin	Golden lion tamarin	*Leontopithecus rosalia*	49	W gibbon	Western black-crested gibbon	*Nomascus concolor*
25	Gorilla	Western Gorilla	*Gorilla gorilla*	50	Wolfi	Wolf’s monkey	*Cercopithecus wolfi*
**B. Carnivore Species**							
1	A bear	American black bear	*Ursus americanus*	22	Lion	Lion	*Panthera leo*
2	A dog	African hunting dog	*Lycaon pictus*	23	M wolf	Maned wolf	*Chrysocyon brachyurus*
3	A fox	Artic fox	*Alopex lagopus*	24	Marten	European pine marten	*Martes martes*
4	B bear	Brown bear	*Ursus arctos*	25	Meerkat	Meerkat	*Suricata suricatta*
5	B dog	Bush dog	*Speothos venaticus*	26	Mongoose	Common dwarf mongoose	*Helogale parvula*
6	B fox	Bat-eared fox	*Otocyon megalotis*	27	O otter	Oriental small-clawed otter	*Amblonyx cinerea*
7	Binturong	Binturong	*Arctictis binturong*	28	Ocelot	Ocelot	*Leopardus pardalis*
8	C fox	Corsac fox	*Vulpes corsac*	29	P bear	Polar bear	*Ursus maritimus*
9	C leopard	Clouded leopard	*Neofelis nebulosa*	30	R panda	Red panda	*Ailurus fulgens*
10	C lynx	Canadian lynx	*Lynx canadensis*	31	Racoon	Raccoon	*Procyon lotor*
11	Cheetah	Cheetah	*Acinonyx jubatus*	32	Re. fox	Red fox	*Vulpes vulpes*
12	Coati	South American coati	*Nasua nasua*	33	Ru. fox	Rüppell’s fox	*Vulpes rueppellii*
13	Cougar	Cougar	*Puma concolor*	34	S leopard	Snow leopard	*Uncia uncia*
14	Dhole	Dhole	*Cuon alpinus*	35	Sea lion	California sea lion	*Zalophus californianus*
15	E lynx	Eurasian Lynx	*Lynx lynx*	36	Serval	Serval	*Leptailurus serval*
16	E otter	European otter	*Lutra lutra*	37	Sp. bear	Spectacled bear	*Tremarctos ornatus*
17	E wolf	Eurasian wolf	*Canis lupus*	38	Stoat	Stoat	*Mustela ermine*
18	Hyena	Striped hyena	*Hyaena hyaena*	39	Su. Bear	Sun bear	*Helarctos malayanus*
19	Jaguar	Jaguar	*Panthera onca*	40	Tiger	Bengal tiger	*Pathera tigris tigris*
20	Jaguarundi	Jaguarundi	*Herpailurus yagouaroundi*	41	Weasel	Least weasel	*Mustela nivalis*
21	Kinkajou	Kinkajou	*Potos flavus*				

**Table 2:** Statistics for LogBL vs. LogDF10 and LogDF6 comparisons.

Model	*Slope* ± *SE*	*Intercept* ± *SE*	*R*^*2*^	*λ*	*t*	*d.f.*	*P*
**A. LogBL vs. logDF10**							
**PRIMATES (n = 50)**							
OLS	−1.722 ± 0.173	6.149 ± 0.288	0.673	—	21.34	48	< 0.0001
Y-on-X (BM)	−0.831 ± 0.286	4.666 ± 0.551	—	—	−2.9	48	0.0056
X-on-Y (BM)	−5.568 ± 0.062	12.606 ± 0.239	—	—	−2.9	48	0.0056
**CARNIVORES (n** = **41)**							
OLS	−0.679 ± 0.166	4.395 ± 0.32	0.299	—	−4.08	39	0.0002
Y-on-X (NP)	−0.679 ± 0.166	4.395 ± 0.32	—	—	−4.08	39	0.0002
X-on-Y (NP)	−2.267 ± 0.108	7.425 ± 0.337	—	—	−4.08	39	0.0002
**COMBINED (n** = **91)**							
OLS	−1.010 ± 0.113	4.997 ± 0.203	0.471	—	24.65	89	<0.0001
Y-on-X (BM)	−0.685 ± 0.186	4.452 ± 0.43	—	—	−3.69	89	0.0004
X-on-Y (BM)	−5.157 ± 0.053	12.502 ± 0.222	—	—	−3.69	89	0.0004
**B. LogBL vs. logDF6**							
**PRIMATES (n = 41)**							
OLS	−1.653 ± 0.197	6.069 ± 0.326	0.644	—	18.62	39	<0.0001
Y-on-X (BM + λ)	−1.15 ± 0.364	5.209 ± 0.649	—	0.917	−3.16	39	0.003
X-on-Y (BM + λ)	−5.913 ± 0.057	13.147 ± 0.21	—	0.938	−2.98	39	0.0049
**CARNIVORES (n = 33)**							
OLS	−0.652 ± 0.186	4.384 ± 0.356	0.284	—	12.33	31	<0.0001
Y-on-X (NP)	−0.652 ± 0.186	4.384 ± 0.356	—	—	12.33	31	<0.0001
X-on-Y (NP)	−2.298 ± 0.124	7.496 ± 0.394	—	—	−3.5	31	0.0014
**COMBINED (n = 74)**							
OLS	−0.959 ± 0.126	4.943 ± 0.224	0.445	—	22.08	72	<0.0001
Y-on-X (BM + λ)	−0.671 ± 0.21	4.443 ± 0.437	—	0.953	−3.19	72	0.0021
X-on-Y (BM + λ)	−5.921 ± 0.053	13.88 ± 0.222	—	0.996	−2.98	72	0.0039

Y-on-X and X-on-Y refer to the two regressions (Y-dependent-on-X and X-dependent-on-Y) from which bisectors were calculated (X = logBL in A & B, Y = logDF10 in A and logDF6 in B); abbreviations in parentheses specify the best-supported GLS model (BM = Brownian motion, NP = non-phylogenetic, BM + λ = Brownian motion + Pagel’s lambda). For the BM + λ models, λ describes the extent to which the dependent variable covaries with phylogeny. T-tests assess whether regression slopes are significantly different than 0. Regression intercepts were all significantly different from 0 (p < 0.0001).

**Table 3:** Statistics for LogBL vs. LogF06 comparisons.

Model	*Slope* ± *SE*	*Intercept* ± *SE*	*R*^*2*^	*λ*	*t*	*d.f.*	*P*
**PRIMATES (n = 41)**							
OLS	−2.456 ± 0.233	7.177 ± 0.385	0.741	—	18.64	39	<0.0001
Y-on-X (BM)	−1.681 ± 0.467	5.902 ± 0.869	—	—	−3.6	39	0.0009
X-on-Y (BM)	−6.471 ± 0.041	14.347 ± 0.171	—	—	−3.6	39	0.0009
**CARNIVORES (n** = **33)**							
Model	*Slope* ± *SE*	*Intercept* ± *SE*	*R*^*2*^	*λ*	*t*	*d.f.*	*P*
OLS	−1.004 ± 0.202	4.695 ± 0.386	0.443	—	−4.97	31	<0.0001
Y-on-X (NP)	−1.004 ± 0.202	4.695 ± 0.386	0.443	—	−4.97	31	<0.0001
X-on-Y (NP)	−2.265 ± 0.089	7.078 ± 0.251	0.443	—	−4.97	31	<0.0001
**COMBINED (n = 74)**							
Model	*Slope* ± *SE*	*Intercept* ± *SE*	*R*^*2*^	*λ*	*t*	*d.f.*	*P*
OLS	−1.483 ± 0.149	5.588 ± 0.265	0.579	—	−9.949	72	<0.0001
Y-on-X (BM + λ)	−1.179 ± 0.227	5.06 ± 0.451	—	0.795	−5.19	72	<0.0001
X-on-Y (BM + λ)	−5.441 ± 0.043	12.719 ± 0.181	—	0.989	−4.26	72	<0.0001

Format is the same as Table 2 (X = logBL, Y = logF06).
