# Supplementary material for: Body size and vocalization in primates and carnivores
Source: Sci Rep. 2017 Jan 24;7:41070. doi: 10.1038/srep41070 (PMC5259760; doi:10.1038/srep41070)
Supplement: Supplementary Information [file srep41070-s1.pdf]

Body size and vocalizations in primates and carnivores

Bowling DL, Garcia M, Dunn JC, Ruprecht R, Stewart A, Frommolt K-H, Fitch WT.

SUPPLEMENTARY MATERIALS

Supplementary Text S1. Bisector regressions

Supplementary Figure S1. Primates phylogenetic tree

Supplementary Figure S2. Carnivores phylogenetic tree

Supplementary Figure S3. LogBL vs. logDF6

Supplementary Table S1. GLS models for LogBL vs. logDF10 & LogBL vs. logDF6

Supplementary Table S2. GLS models for LogBL vs. logF06

Supplementary Table S3. Data for all comparisons

Supplementary Text S1. Bisector regressions

The bisector regressions combine phylogenetic regression techniques and bisector regression techniques to address limitations of ordinary least squares (OLS) regression in interspecific size-frequency allometry. Phylogenetic regression accounts for the potential non-independence of data points due to shared phylogenetic history by incorporating expectations about residual covariance based on phylogenetic branch length [1]. Branch lengths were derived from consensus trees for primates and carnivores downloaded from the 10kTrees project website on January 14, 2016 [2] (Supplementary Figs. S1 & S2). For combined analyses, the primate and carnivore trees were joined using a splitting date of 87.8 million years ago [3].

Following Charlton & Reby (2016), the generalized least squares (GLS) regression models on which the bisector regressions were based included: (1) a non-phylogenetic model (NP), which is essentially equivalent to OLS; (2) a pure Brownian motion model (BM), in which trait differences between two species are assumed to be proportional to the amount of time since their split from a common ancestor; (3) a Brownian motion + Pagel's Lambda model (BM+λ), which additionally accounts for the extent to which trait values actually covary with phylogeny (λ=0 indicates phylogenetic independence and is equivalent to NP or OLS, λ=1 is equivalent to pure BM); (4) a Brownian motion + Grafen's Rho model (BM+ρ), which tests for differences in the rate of evolutionary change (ρ<1 indicates relatively more recent gradual evolution; ρ>1 indicates faster recent evolution); and (5) an Ornstein-Uhlenbeck (OU) model which uses a parameter called α to test the strength of stabilizing selection towards an optimum (α =0 is equivalent to pure BM, higher values of α indicate stronger stabilizing selection) [4]. The

optimum values of the parameters λ, ρ, α were estimated for their respective models in R by maximizing the restricted log-likelihood (‘method’ argument of nlme package function ‘gls’ set to ‘REML’) [5].

Bisector regression is appropriate when it is not clear which variable in a comparison is ‘independent’ and which is ‘dependent’ [6]. In all of the allometric comparisons made here, neither variable was truly independent because both were measured and thus subject to deviation/error. Bisector regression accounts for error in both variables by calculating the line that bisects the minor angle between two separate regressions: one based on minimizing error with respect to the Y variable (the ‘Y-on-X’ regression) and one based on minimizing error with respect to the X variable (the ‘X-on-Y’ regression) [6,7].

For each of the comparisons presented in Figures 2, 3 and Supplementary Figure S3, bisector regressions were calculated in four steps. First, which the 5 different regression models (NP, BM, BM+λ , BM+ρ and OU) had the lowest AICc value for the Y-on-X regression was determined. Second, the X-on-Y regression for this model was calculated (by reversing the position of the variables in the regression formula). Third, this X-on-Y regression was transposed into the same coordinate space as the Y-on-X regressions by solving for the x-intercept (which became the y-intercept) and inverting the slope. And fourth, finding the line that passes through the intersection of the Y-on-X and transposed X-on-Y lines, with a slope determined by the formula:

$$\tan(\ (\ \arctan(mYX) + \arctan(mXYt)\ )\ /2\ )$$

where mYX is the slope of the Y-on-X line, and mXYt is the slope of the inverted X-on-Y line. These operations were carried out in Matlab using the function ‘polyxpoly.m’ to

calculate the point of intersection, and the functions ‘tan.m’ and ‘atan.m’ to determine the slope.

Supplementary Text S1 References

1. Symonds, M. R. E. & Blomberg, S. P. in *Modern phylogenetic comparative methods* (ed. Garamszegi, L. Z.) 105–130 (Springer, 2014).
2. Arnold, C., Matthews, L. J. & Nunn, C. L. The 10kTrees website: a new online resource for primate phylogeny. *Evol. Anthropol.* **19,** 114–118 (2010).
3. dos Reis, M. *et al.* Phylogenomic datasets provide both precision and accuracy in estimating the timescale of placental mammal phylogeny. *Proc. R. Soc. B Biol. Sci.* **279,** 3491–3500 (2012).
4. Charlton, B. D. & Reby, D. The evolution of acoustic size exaggeration in terrestrial mammals. *Nat. Commun.* **7,** 12739 (2016).
5. Pinheiro, J., Bates, D., DebRoy, S., Sarkar, D. & Team, R. C. nlme: linear and nonlinear mixed effects models. (2016).
6. Isobe, T., Feigelson, E. D., Akritas, M. G. & Babu, G. J. Linear regression in astromony. I. *Astrophys. J.* **364,** 104–113 (1990).
7. Sprent, P. & Dolby, G. R. The geometric mean functional relationship. *Biometrics* **36,** 547–550 (1980).

Supplementary Figure S1. Primates phylogenetic tree

Selected species are shown in red

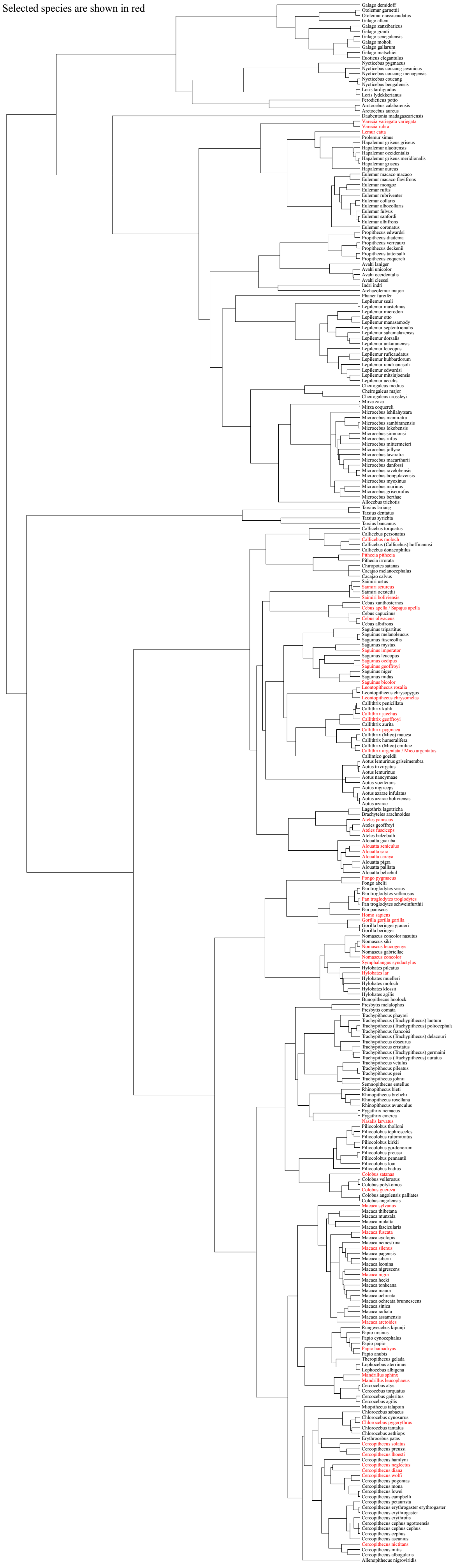

Supplementary Figure S2. Carnivores phylogenetic tree

Selected species are shown in red

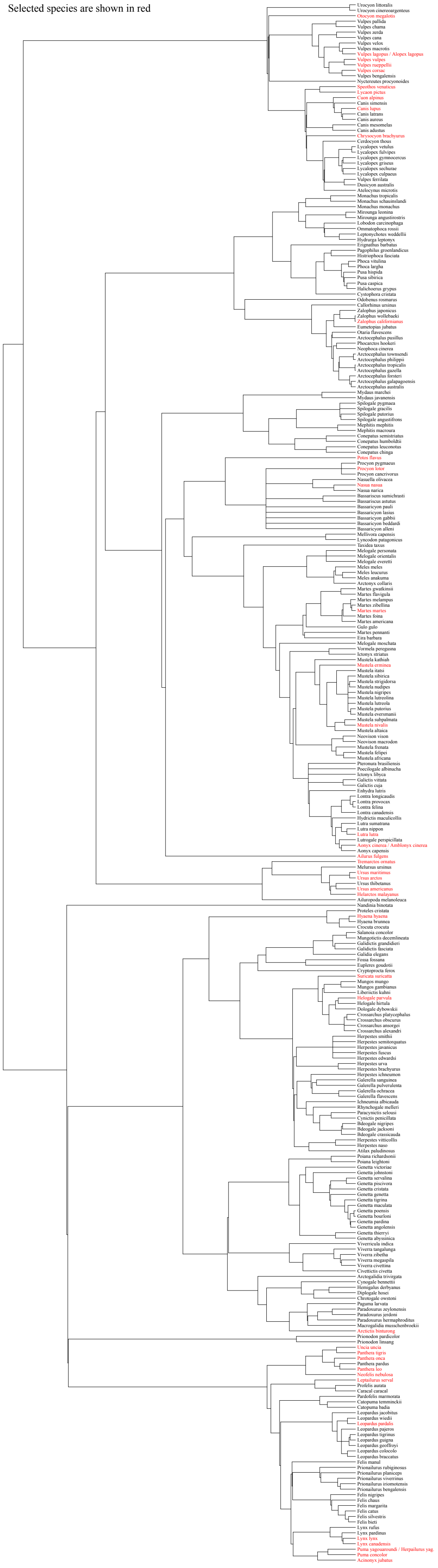

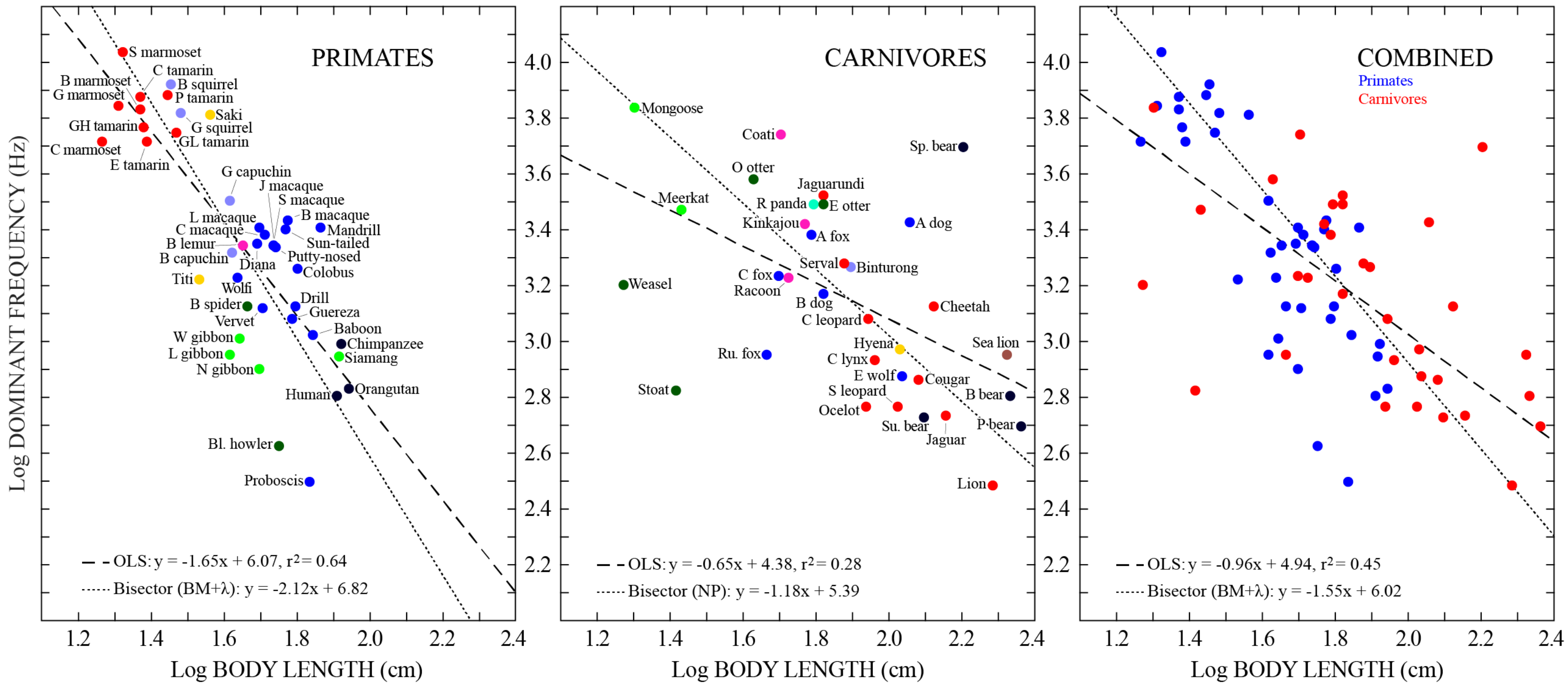

Supplementary Figure S3. Body length and dominant frequency. The base-10 logarithm of mean body length plotted against the base-10 logarithm of mean dominant frequency ('DF6') for 41 primate species (left), 33 carnivore species (middle), and all 74 species combined (right). These species correspond to the subset for which F06 values could also be calculated, making these results directly comparable with those in Figure 3. In the left and middle panels, color represents biological family (legend in Figure 4) and each species is labeled with an abbreviated form of its common name (full names in Table 1); in the right panel primates are shown in blue and carnivores in red. Dashed lines depict ordinary least squares (OLS) regressions; dotted lines depict bisector regressions (equations at lower left). Primate and combined bisector regressions were based on phylogenetic Brownian motion + Pagel's Lambda (BM+λ) models; carnivore bisector regressions were based on non-phylogenetic (NP) models. Statistics for regression analyses are given in Table 2b (see Supplementary Table S1b for AICc values of GLS models).

Supplementary Table S1a. GLS models for logBL vs. logDF10

**PRIMATES** (n = 50)						
Model	*Slope ± SE*	*Intercept ± SE*	*λ*	ρ	α	*AICc*
BM	-0.831 ± 0.286 **	4.666 ± 0.551 ****	-	-	-	-13.926
BM+λ	-0.887 ± 0.287 **	4.76 ± 0.547 ****	0.992	-	-	-11.806
BM+ρ	-1.338 ± 0.496 ****	5.501 ± 0.496 ****	-	0.27	-	0.588
NP	-1.722 ± 0.173 ****	6.149 ± 0.288 ****	-	-	-	7.926
OU	-1.722 ± 0.173 ****	6.149 ± 0.288 ****	-	-	1	10.293
**CARNIVORES** (n = 41)						
Model	*Slope ± SE*	*Intercept ± SE*	*λ*	ρ	α	*AICc*
NP	-0.679 ± 0.166 ***	4.395 ± 0.32 ****	-	-	-	24.792
BM+ρ	-0.641 ± 0.18 **	4.326 ± 0.35 ****	-	0.051	-	26.854
BM+λ	-0.654 ± 0.173 ***	4.355 ± 0.333 ****	0.053	-	-	27.135
OU	-0.678 ± 0.166 ***	4.395 ± 0.32 ****	-	-	1	27.254
BM	-0.629 ± 0.265 **	4.372 ± 0.578 ****	-	-	-	31.677
**COMBINED** (n = 91)						
Model	*Slope ± SE*	*Intercept ± SE*	*λ*	ρ	α	*AICc*
BM	-0.685 ± 0.186 ***	4.452 ± 0.43 ****	-	-	-	18.073
BM+λ	-0.704 ± 0.185 ***	4.485 ± 0.415 ****	0.983	-	-	19.279
BM+ρ	-0.782 ± 0.16 ****	4.59 ± 0.312 ****	-	0.25	-	26.923
NP	-1.01 ± 0.113 ****	4.997 ± 0.203 ****	-	-	-	41.046
OU	-1.01 ± 0.113 ****	4.997 ± 0.203 ****	-	-	1	43.235

Supplementary Table S1b. GLS models for logBL vs. logDF6

**PRIMATES** (n = 41)						
Model	*Slope ± SE*	*Intercept ± SE*	*λ*	ρ	α	*AICc*
BM+λ	-1.15 ± 0.364 **	5.209 ± 0649 ****	0.917	-	-	3.071
BM	-0.871 ± 0.385 *	4.741 ± 0.716 ****	-	-	-	3.604
BM+ρ	-1.509 ± 0.296 ****	5.816 ± 0.494 ****	-	0.12	-	6.986
NP	-1.653 ± 0.197 ****	6.069 ± 0.326 ****	-	-	-	7.355
OU	-1.653 ± 0.197 ****	6.069 ± 0.326 ****	-	-	1	9.818
**CARNIVORES** (n = 33)						
Model	*Slope ± SE*	*Intercept ± SE*	*λ*	ρ	α	*AICc*
NP	-0.652 ± 0.186 **	4.384 ± 0.356 ****	-	-	-	24.499
BM+λ	-0.66 ± 0.184 **	4.397 ± 0.351 ****	-0.018	-	-	27.086
BM+ρ	-0.652 ± 0.186 **	4.383 ± 0.356 ****	-	0.0003	-	27.1
OU	-0.652 ± 0.186 **	4.384 ± 0.356 ****	-	-	1	27.1
BM	-0.572 ± 0.291*	4.287 ± 0.618 ****	-	-	-	30.486
**COMBINED** (n = 74)						
Model	*Slope ± SE*	*Intercept ± SE*	*λ*	ρ	α	*AICc*
BM+λ	-0.671 ± 0.21 **	4.443 ± 0.437 ****	0.935	-	-	30.182
BM	-0.65 ± 0.219 **	4.406 ± 0.485 ****	-	-	-	30.231
BM+ρ	-0.78 ± 0.166 ****	4.62 ± 0.309 ****	-	0.153	-	31.347
NP	-0.959 ± 0.126 ****	4.943 ± 0.224 ****	-	-	-	35.424
OU	-0.959 ± 0.126 ****	4.943 ± 0.224 ****	-	-	1	37.660

The 5 general least squares regression models used to assess each relationship between body length and dominant frequency examined here, ranked by Akaike Information Criterion corrected for sample size (AICc). NP = non-phylogenetic, BM = Brownian motion, BM+λ = Brownian motion + Pagel's lambda, BM+ρ = Brownain motion + Grafen's rho, OU = Ornstein-Uhlenbeck. **** p < 0.0001, *** p < 0.001, ** p <0.01, * p<0.05

Supplementary Table S2. GLS models for logBL vs. logDF6

**PRIMATES** (n = 41)						
Model	*Slope ± SE*	*Intercept ± SE*	λ	ρ	α	*AICc*
BM	-1.681 ± 0.467 **	5.902 ± 0.869 ***	-	-	-	18.714
BM+λ	-1.845 ± 0.449 **	6.177 ± 0.801 ***	0.913	-	-	19.8
NP	-2.456 ± 0.232 ***	7.177 ± 0.385 ***	-	-	-	20.318
BM+ρ	-2.234 ± 0.343 ***	6.811 ± 0.572 ***	-	0.102	-	21.059
OU	-2.456 ± 0.232 ***	7.177 ± 0.385 ***	-	-	1	22.78
**CARNIVORES** (n = 33)						
Model	*Slope ± SE*	*Intercept ± SE*	λ	ρ	α	*AICc*
NP	-1.004 ± 0.202 ***	4.695 ± 0.386 ***	-	-	-	29.612
BM+ρ	-0.996 ± 0.206 ***	4.679 ± 0.394 ***	-	0.013	-	32.167
BM+λ	-1.003 ± 0.202 ***	4.693 ± 0.387 ***	0.002	-	-	32.212
OU	-1.004 ± 0.202 ***	4.695 ± 0.386 ***	-	-	1	32.213
BM	-0.873 ± 0.331	4.469 ± 0.704 ***	-	-	-	38.588
**COMBINED** (n = 74)						
Model	*Slope ± SE*	*Intercept ± SE*	λ	ρ	α	*AICc*
BM+λ	-1.179 ± 0.227 ***	5.06 ± 0.451 ***	0.795	-	-	51.429
BM+ρ	-1.238 ± 0.9 ***	5.152 ± 0.353 ***	-	0.144	-	51.903
BM	-1.09 ± 0.26 **	4.9 ± 0.575 ***	-	-	-	54.775
NP	-1.483 ± 0.149 ***	5.588 ± 0.265 ***	-	-	-	59.454
OU	-1.483 ± 0.149 ***	5.588 ± 0.265 ***	-	-	1	61.691

Format same as Table S1.

Supplementary Table 3a. Primate data for all comparisons

PRIMATES

Number	Order	Family	Latin Name	Common Name	Abbreviation	BL (cm)	DF10 (Hz)	DF6 (Hz)	F06 (Hz)	Number of available vocalizations after preprocessing	Number of non-tonal vocalizations (out of 10)
p01	Primates	Cercopithecidae	Allochrocebus lhoesti	L'Hoest's monkey	L'Hoest	56	2464			65	10
p02	Primates	Cercopithecidae	Allochrocebus solatus	Sun-tailed monkey	Sun-tailed	58.75	2487	2535.333	400.333	117	4
p03	Primates	Atelidae	Alouatta caraya	Black howler	Bl. howler	56.25	437	424.5	241.667	23	0
p04	Primates	Atelidae	Alouatta sara	Bolivian red howler	Bo. howler	62.6	322			26	6
p05	Primates	Atelidae	Alouatta seniculus	Colombian red howler	C howler	54.75	503			37	8
p06	Primates	Atelidae	Ateles fusciceps	Black-headed spider monkey	B spider	46.25	1230	1338	1092	21	0
p07	Primates	Atelidae	Ateles paniscus	Red-faced spider monkey	R spider	54.375	1779			61	5
p08	Primates	Pitheciidae	Callicebus moloch	Red-bellied titi	Titi	34.15	1754	1672.833	1300.17	90	1
p09	Primates	Callitrichidae	Callithrix flaviceps	Buffy-headed marmoset	B marmoset	23.5	6969	6793.5	5805	172	0
p10	Primates	Callitrichidae	Callithrix geoffroyi	Geoffroy's tufted-ear marmoset	G marmoset	20.5	6943	6945.167	7093.67	42	0
p11	Primates	Callitrichidae	Callithrix jacchus	Common Marmoset	C marmoset	18.5	4953	5228	7179.83	163	4
p12	Primates	Callitrichidae	Cebuella pygmaea	Pygmy marmoset	P marmoset	14	10918			52	5
p13	Primates	Cebidae	Cebus olivaceus	Guianan weeper capuchin	G capuchin	41.5	2975	3174.5	2862.67	42	2
p14	Primates	Cercopithecidae	Cercopithecus diana	Diana monkey	Diana	49.25	2487	2229.333	1257.67	85	4
p15	Primates	Cercopithecidae	Cercopithecus neglectus	De Brazza's monkey	De Brazza	49	2085			145	6
p16	Primates	Cercopithecidae	Cercopithecus nictitans	Putty-nosed monkey	Putty-nosed	55.25	2029	2181.167	201.167	44	2
p17	Primates	Cercopithecidae	Cercopithecus wolfi	Wolf's monkey	Wolf	43.5	1641	1700.833	394.333	23	0
p18	Primates	Cercopithecidae	Chlorocebus pygerythrus	Vervet monkey	Vervet	51	1291	1312	642.167	223	2
p19	Primates	Cercopithecidae	Colobus guereza	Mantled guereza	Guereza	61.2	1067	1208.833	580	72	1
p20	Primates	Cercopithecidae	Colobus satanas	Black colobus	Colobus	63.5	1317	1832.167	848.667	188	1
p21	Primates	Hominidae	Gorilla gorilla	Western Gorilla	Gorilla	105	586			155	7
p22	Primates	Hominidae	Homo sapiens	Human	Human	81.35	577	635.1667	254.333	130	0
p23	Primates	Hylobatidae	Hylobates lar	Lar gibbon	L gibbon	41.5	900	901.3333	867	208	0
p24	Primates	Lemuridae	Lemur catta	Ring-tailed lemur	Ri. Lemur	42.5	3390			118	8
p25	Primates	Callitrichidae	Leontopithecus chrysomelas	Golden-headed lion tamarin	GH tamarin	24	5619	5877	6494	50	0
p26	Primates	Callitrichidae	Leontopithecus rosalia	Golden lion tamarin	GL tamarin	29.5	5448	5628.833	5414	311	0
p27	Primates	Cercopithecidae	Macaca arctoides	Stump-tailed macaque	S macaque	54.55	2138	2194.833	1729.17	26	3
p28	Primates	Cercopithecidae	Macaca fuscata	Japanese macaque	J macaque	54.675	2021	2212.5	1248.67	148	0
p29	Primates	Cercopithecidae	Macaca nigra	Crested macaque	C macaque	51.625	2285	2403.5	1234.17	77	3
p30	Primates	Cercopithecidae	Macaca silenus	Lion-tailed macaque	L macaque	50	2305	2567.667	1101	105	3
p31	Primates	Cercopithecidae	Macaca sylvanus	Barbary macaque	B macaque	59.55	2617	2707.5	1928.67	84	1
p32	Primates	Cercopithecidae	Mandrillus leucophaeus	Drill	Drill	62.625	1231	1331.5	315.333	118	4
p33	Primates	Cercopithecidae	Mandrillus sphinx	Mandrill	Mandrill	73.5	2022	2559.667	355.333	138	3
p34	Primates	Callitrichidae	Mico argentatus	Silvery marmoset	S marmoset	21	10991	10931.83	10880.2	36	0
p35	Primates	Cercopithecidae	Nasalis larvatus	Proboscis monkey	Proboscis	68.5	294	312	114	51	0
p36	Primates	Hylobatidae	Nomascus concolor	Western black-crested gibbon	W gibbon	44	992	1019	1065.83	824	0
p37	Primates	Hylobatidae	Nomascus leucogenys	Northern white-cheecked gibbon	N gibbon	49.75	801	794.8333	812.167	131	0
p38	Primates	Hominidae	Pan troglodytes	Chimpanzee	Chimpanzee	83.5	938	974.1667	700.833	155	0
p39	Primates	Cercopithecidae	Papio hamadryas	Hamadryas baboon	Baboon	70	1011	1051.667	591.5	150	3
p40	Primates	Pitheciidae	Pithecia pithecia	White-faced saki	Saki	36.575	6608	6530.833	6439.17	57	4
p41	Primates	Hominidae	Pongo pygmaeus	Orangutan	Orangutan	87.5	580	678	409.333	106	1
p42	Primates	Callitrichidae	Saguinus bicolor	Pied tamarin	P tamarin	28	7740	7678	7447.5	21	0
p43	Primates	Callitrichidae	Saguinus imperator	Emperor tamarin	E tamarin	24.5	5371	5188.333	5666.5	115	1
p44	Primates	Callitrichidae	Saguinus oedipus	Cotton-top tamarin	C tamarin	23.5	7051	7512.833	5784	197	0
p45	Primates	Cebidae	Saimiri boliviensis	Black-capped squirrel monkey	B squirrel	28.5	8183	8312	7920	29	3
p46	Primates	Cebidae	Saimiri sciureus	Guianan squirrel monkey	G squirrel	30.25	6552	6624.667	5530.83	122	2
p47	Primates	Cebidae	Sapajus apella	Brown capuchin	B capuchin	42	2016	2091.333	2127	197	0
p48	Primates	Hylobatidae	Symphalangus syndactylus	Siamang	Siamang	82.5	813	880.8333	553.5	513	0
p49	Primates	Lemuridae	Varecia rubra	Red-ruffed lemur	Re. lemur	52.5	1421			27	6
p50	Primates	Lemuridae	Varecia variegata	Black-and-white ruffed lemur	B lemur	45	1877	2217	1436.33	224	3

Supplementary Table 3a. Carnivore data for all comparisons

CARNIVORES											
Number	Order	Family	Latin Name	Common Name	Abbreviation	BL (cm)	DF10 (Hz)	DF6 (Hz)	F06 (Hz)	Number of available vocalizations after preprocessing	Number of non-tonal vocalizations (out of 10)
c01	Carnivora	Felidae	Acinonyx jubatus	Cheetah	Cheetah	133	1325	1330.167	1155	52	2
c02	Carnivora	Ailuridae	Ailurus fulgens	Red panda	R panda	62	2953	3119.5	1778.17	208	2
c03	Carnivora	Canidae	Alopex lagopus	Artic fox	A fox	61.25	2195	2418.167	664.833	64	4
c04	Carnivora	Mustelidae	Amblonyx cinerea	Oriental small-clawed otter	O otter	42.5	3718	3800	887.5	286	1
c05	Carnivora	Viverridae	Arctictis binturong	Binturong	Binturong	78.75	1822	1852.833	536.333	59	3
c06	Carnivora	Canidae	Canis lupus	Eurasian wolf	E wolf	108.5	685	749.1667	449.5	109	2
c07	Carnivora	Canidae	Chrysocyon brachyurus	Maned wolf	M wolf	105	849			69	7
c08	Carnivora	Canidae	Cuon alpinus	Dhole	Dhole	111.75	2522			48	6
c09	Carnivora	Ursidae	Helarctos malayanus	Sun bear	Su. bear	125	529	536.3333	252.167	76	4
c10	Carnivora	Herpestidae	Helogale parvula	Common dwarf mongoose	Mongoose	20.05	6878	6915.5	6377.83	55	1
c11	Carnivora	Felidae	Herpailurus yagouaroundi	Jaguarundi	Jaguarundi	66	3374	3352.833	2189.17	24	0
c12	Carnivora	Hyaenidae	Hyaena hyaena	Striped hyena	Hyena	107.5	934	940.6667	167	41	1
c13	Carnivora	Felidae	Leopardus pardalis	Ocelot	Ocelot	86.3	567	580.3333	207.667	61	4
c14	Carnivora	Felidae	Leptailurus serval	Serval	Serval	75.5	1929	1911.833	405.167	106	4
c15	Carnivora	Mustelidae	Lutra lutra	European otter	E otter	66	2872	3088.5	1010.67	103	0
c16	Carnivora	Canidae	Lycaon pictus	African hunting dog	A dog	114.25	2555	2654.833	2073.17	120	2
c17	Carnivora	Felidae	Lynx canadensis	Canadian lynx	C lynx	91.45	857	856.1667	417.667	36	0
c18	Carnivora	Felidae	Lynx lynx	Eurasian Lynx	E lynx	95	617			79	5
c19	Carnivora	Mustelidae	Martes martes	European pine marten	Marten	51.5	1571			29	7
c20	Carnivora	Mustelidae	Mustela erminea	Stoat	Stoat	26.125	655	663.3333	609.833	38	2
c21	Carnivora	Mustelidae	Mustela nivalis	Least weasel	Weasel	18.7	1611	1583.167	1382.33	25	3
c22	Carnivora	Procyonidae	Nasua nasua	South American coati	Coati	50.5	5480	5502.333	5045.33	247	3
c23	Carnivora	Felidae	Neofelis nebulosa	Clouded leopard	C leopard	87.65	1165	1211.5	395.333	32	2
c24	Carnivora	Canidae	Otocyon megalotis	Bat-eared fox	B fox	53.575	1354			45	9
c25	Carnivora	Felidae	Panthera leo	Lion	Lion	193	285	303.3333	172.167	171	4
c26	Carnivora	Felidae	Panthera onca	Jaguar	Jaguar	143	485	540.6667	116.667	141	4
c27	Carnivora	Felidae	Panthera tigris	Bengal tiger	Tiger	218	321			93	5
c28	Carnivora	Procyonidae	Potos flavus	Kinkajou	Kinkajou	59	2601	2620.5	2090.17	93	2
c29	Carnivora	Procyonidae	Procyon lotor	Raccoon	Racoon	53	1583	1690.833	749.167	106	3
c30	Carnivora	Felidae	Puma concolor	Cougar	Cougar	120.5	660	730.6667	195	76	4
c31	Carnivora	Canidae	Speothos venaticus	Bush dog	B dog	66.25	1345	1486.167	780	71	0
c32	Carnivora	Herpestidae	Suricata suricatta	Meerkat	Meerkat	27	2059	2965.167	1312.67	84	3
c33	Carnivora	Ursidae	Tremarctos ornatus	Spectacled bear	Sp. bear	160	4662	4977.333	543.333	109	1
c34	Carnivora	Felidae	Uncia uncia	Snow leopard	S leopard	105.5	559	585.3333	336.667	70	4
c35	Carnivora	Ursidae	Ursus americanus	American black bear	A bear	155	824			26	6
c36	Carnivora	Ursidae	Ursus arctos	Brown bear	B bear	215	629	635.3333	187.5	93	2
c37	Carnivora	Ursidae	Ursus maritimus	Polar bear	P bear	230	431	499.5	199.333	40	4
c38	Carnivora	Canidae	Vulpes corsac	Corsac fox	C fox	49.875	1689	1714.167	755.833	87	4
c39	Carnivora	Canidae	Vulpes rueppellii	Rüppell's fox	Ru. fox	46.35	849	893.3333	822.5	43	3
c40	Carnivora	Canidae	Vulpes vulpes	Red fox	Re. fox	65.5	927			43	6
c41	Carnivora	Otariidae	Zalophus californianus	California sea lion	Sea lion	210	848	891.3333	349.167	112	1
